# CXCR3 Ligands in Cancer and Autoimmunity, Chemoattraction of Effector T Cells, and Beyond

**DOI:** 10.3389/fimmu.2020.00976

**Published:** 2020-05-29

**Authors:** Nathan Karin

**Affiliations:** Department of Immunology, Faculty of Medicine, Technion- Israel Institute of Technology, Haifa, Israel

**Keywords:** CXCR3, chemokines, CXCL10, CXCL9, EAE, cancer, tolerance

## Abstract

CXCR3 is a chemokine receptor with three ligands; CXCL9, CXCL10, and CXCL11. CXCL11 binds CXCR3 with a higher affinity than the other ligands leading to receptor internalization. Long ago we reported that one of these chemokines, CXCL10, not only attracts CXCR3+ CD4+ and CD8+ effector T cells to sites of inflammation, but also direct their polarization into highly potent effector T cells. Later we showed that CXCL11 directs the linage development of T-regulatory-1 cells (Tr1). We also observed that CXCL11 and CXCL10 induce different signaling cascades via CXCR3. Collectively this suggests that CXCR3 ligands differentially regulate the biological function of T cells via biased signaling. It is generally accepted that tumor cells evolved to express several chemokine receptors and secrete their ligands. Vast majority of these chemokines support tumor growth by different mechanisms that are discussed. We suggest that CXCL10 and possibly CXCL9 differ from other chemokines by their ability to restrain tumor growth and enhance anti-tumor immunity. Along with this an accumulating number of studies showed in various human cancers a clear association between poor prognosis and low expression of CXCL10 at tumor sites, and vice versa. Finally, we discuss the possibility that CXCL9 and CXCL10 may differ in their biological function via biased signaling and its possible relevance to cancer immunotherapy. The current mini review focuses on exploring the role of CXCR3 ligands in directing the biological properties of CD4+ and CD8+ T cells in the context of cancer and autoimmunity. We believe that the combined role of these chemokines in attracting T cells and also directing their biological properties makes them key drivers of immune function.

## Introduction

Chemokines are small (~8–14 kDa), structurally cytokine-like, secreted proteins that regulate cell trafficking through interactions with a subset of 20 different seven-transmembrane, G protein-coupled receptors (GPCRs) (1). These receptors could be divided into single (mono) receptors, and shared receptors in which a single receptor binds several chemokines. Different chemokines that bind a shared receptor may have different modes of interactions. They may either poses similar biological properties (may explain redundancy), or induce divers signaling cascades and thereby differ in biological properties. This type of biased signaling has been previously observed for beta2-adrenergic receptor (also GPCRs) by the Nobel prize winner Robert J. Lefkowitz (2) and by others (3). Our laboratory was the first to report that such biased signaling is also used by chemokines to direct the biological properties of CD4+ T cells in controlling effector T cell function vs. tolerance to self (4, 5), and perhaps in controlling anti-cancer immunity (6). The current review focuses on the role of CXCR3 and its ligands: CXCL9, CXCL10, and CXCL11 on the biological function of CD4+ and CD8+ T cells and its translational implications.

Of the three CXCR3 ligands most of the attention has been drawn thus far to CXCL10, as a candidate for cancer immunotherapy. Only recently it has been suggested that that CXCL9 is also involved in directing the potentiation of CD8+ T cells in cancer, and that its activity differs from CCL10 (7). Not much is known about the role of CXCL11 in cancer diseases. As for autoimmunity, the role of CXCL10 and CXCL11 has been largely studied by several laboratories including ours, whereas the role of CXCL9 is still elusive (8).

## CXCR3 and Its Ligands

CXCR3 is a chemokine receptor that is primarily expressed on CD4+ and CD8+ T cells, and to some extent by other cells, among them, epithelial cells (9). Within the CD4+ subset CXCR3 is mostly abundant on proinflammatory Th1 cells, but notably it is also expressed by FOXp3+ regulatory T cells (T_regs_) (10–12). Mice express a single isoform of CXCR3 that exclusively bind CXCL9, CXCL10, and CXCL11. In human three isoforms were identified: CXCR3A that is reciprocal to the mouse CXCR3 and also binds CXCL9, CXCL10, and CXCL11, CXCR3-B that binds CXCL9, CXCL10, CXCL11 as well as an additional ligand CXCL4, and CXCR3-alt that only binds CXCL11 (13). The CXCR3 ligands share limited sequence homology. Yet, in their structural homology they are more similar to each other than to other non-ELR chemokines. Also all three chemokines are inducible by IFN-γ (14). Together this makes them a well-characterized subfamily of the non-ELR chemokines. CXCL11 is believed to be the dominant CXCR3 agonist, as it is more potent than CXCL10 or CXCL9 as a chemoattractant and in stimulating calcium flux and receptor desensitization (15).

## Biased Signaling Via CXCR3 Directs the Polarization of CD4+ T Cell Subsets

Based on their cytokine profile FOXp3-negative CD4+ T cells fall into different subsets among them IFN-γ^high^IL4^low^ Th1 cells IFN-γ^low^IL4^high^ Th2 cells, IL17^high^ Th17 cells and IL10^high^ T regulatory-1 (Tr1) cells (16). It is generally accepted that the polarization of non-polarized CD4+ T cells (Thnp) into these subsets is directed by the cytokine milieu within their microenvironment (16). Not much attention has been drawn to the role of chemokines in T cell polarization.

Long ago we observed that along the development of two different experimental autoimmune diseases in Lewis rats: Experimental autoimmune encephalomyelitis (EAE), and adjuvant induced arthritis (AA) the immune system generate an autoantibody response (IgG isotype) to pro-inflammatory cytokines and chemokines that are likely to be involved in the pathogenesis of these diseases (17, 18). In these studies we also observed that amplification of these responses by targeted DNA plasmids may restrain the progression of these diseases (17, 18). We further investigated the mechanistic basis of this response and named it “beneficial autoimmunity” (19). While extending these studies to CXCL10 we learned that targeting the function of CXCL10 restrained the development of EAE or AA. *Ex vivo* analysis of CD4+ T cells subsets indicated for *in vivo* shift from Th1 to Th2 (20, 21). Independently, others observed that CXCL10 promotes the polarization of human CD4+ T cells into IFNγ^high^IL4^low^ Th1 cells (22). The role of CXCL9 in directing effector T cell polarization is yet to be studied. Collectively, this suggests that CXCL10 promotes the polarization of Th1 cells, thus its targeted neutralization restrains autoimmunity. In our studies we could clearly record the effect of CXCL10 neutralization on the Th1/Th2 balance of antigen specific T cells in the periphery (17, 18), and suggested that along the dynamics of each disease these cells are recruited to the inflammatory site, to replace those that undergo apoptosis there (23). The possibility that these antibodies directly enter the CNS to affect T cell polarization there has not been detected.

While further exploring the interplay between CXCR3 ligands, particularly CXCL10 vs. CXCL11 and their role in directing CD4+ T cell polarization we observed that CXCL11 preferentially drives the polarization of IL10^high^ Tr1 cells (4, 5). The underlying signal cascade included signaling via p70 kinase/mTOR in STAT-3- and STAT-6-dependent pathways (4, 5). This differed from CXCL10 that signals via STAT1, STAT4, and STAT5 phosphorylation (4, 5). CXCL11 is believed to be the dominant CXCR3 agonist, as it is more potent than CXCL10 or CXCL9 as a chemoattractant and in stimulating calcium flux and receptor desensitization (15). This suggests that the interplay between CXCL11 and CXCL10 dominates the regulation of CD4+ T cell mediated responses, while favoring active tolerance over effector reactivity. C57BL/6 mice that lack functional CXCL11 due to a shift in the open reading frame of the CXCL11-encoding gene (insertion of two bases after nucleotide 39), resulting in the translation of a chimeric protein lacking the critical CXC motif (24), preferentially induce Th1 oriented response, are highly susceptible to the induction of various Th1-related autoimmune diseases. We observed that these mice are excellent responders to low doses CXCL11-Ig based therapy of EAE in comparison to SJL mice that do not display this open reading frame mutation (4).

The idea of different ligands that differ in their binding site to the same GPCRs receptor also induce different signaling cascade has been primarily investigated by Robert J. Lefkowitz and his team while exploring the Molecular mechanism of beta-arrestin-biased agonism (2, 25, 26). We have explored the relevance of this mechanism for chemokines and T cell regulation.

In summary, we suggest that CXCL11 and CXCL10 plays an opposing role in directing T cell polarization, and as CXCL11 has a higher affinity to CXCR3 it is likely to dominate immune regulation.

## The Contradictive Role of CXCR3-CXCL10 Axis In Neuroinflammation

**Figure 1:**
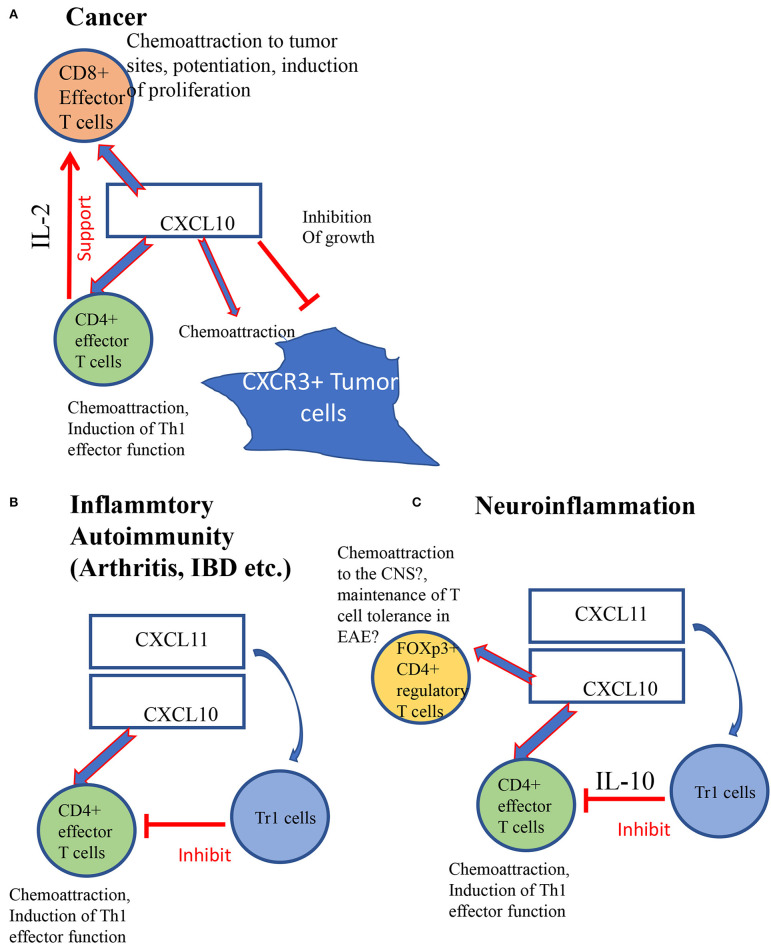
*CXCL10* directs the biological function of CD4+ and CD8+ T cells in cancer and autoimmunity. **(A)** ***The role of CXCL10 in cancer diseases***: CXCL10 directs the accumulation of CXCR3+ effector T cells, in particular effector CD8+ T cells to the tumor site (33) and potentiates their anti-tumor activities, either directly or via the potentiation of effector CD4+ T cells to support their activity. As for tumor cells, it directly suppresses tumor growth (34, 35). Yet, for CNS metastatic spread it had been suggested that CXCL10 produced by astrocytes directs metastatic spread to the brain (36). **(B)** ***The role of CXCL10*** ***and CXCL11 in inflammatory autoimmunity:***CXCL10 is associated with chemoattraction and potentiation of effector T cells that commence the inflammatory process. Its activity is regulated, in part, by CXCL11 that induces T regulatory-1 (Tr1) cells (4). **(C)** Neuroinflammation: In neuroinflammation CXCL10 is likely to hold a duale function. Aside of chemoattraction of effector T cells it selectively induces the accumulation of FOXp3+ Tregs to restrain inflammation (37, 38).

It is largely accepted that CXCL10 promotes the activity of effector CD4+ and CD8+ T cells, and also their recruitment at inflammatory sites (also tumor site) and thus its targeted neutralization could be beneficial in treating various T cell mediated autoimmune diseases among them: psoriasis, rheumatoid arthritis (RA) (27, 28), Inflammatory Bowel Disease [IBD) (29), and type I diabetes (T1DM) (30, 31) (for a recent review also see (32)] (Figure 1B). The role of the CXCL10-CXCR3 axis in neuroinflammation is likely to more complex and controversial (37). The first record that systemic administration of polyclonal antibodies against CXCL10 suppress EAE came from the study of William Karpus and his group in 2001 (39). Independently, and shortly after we reported that targeted DNA vaccines encoding CXCL10 could amplify the production of neutralizing autoantibodies to CXCL10 that could also suppress EAE in Lewis rats (20). Both studies were limited in the use of polyclonal antibodies. Four years later Richard Ransohoff and his group reported that CXCR3 KO mice lacking the CXCR3-CXCL10 interaction develop more severe EAE then WT (40). The absence of CXCR3-CXCL11 interaction could not be taken in account as these were C57BL6 mice lacking functional CXCL11. Klein et al. examined the development of EAE in WT Vs CXCL10 KO mice and observed differences only during sub-optimal induction of disease (41). In another study, Iain Campbell and his group compared the development of EAE in WT and CXCR3KO mice and observed that along the later chronic phase of disease CXCR3KO mice develop a more severe EAE then WT, and that this has been associated with reduced number of FOXp3+ Tregs at the CNS (38). Campbell and his co-authors suggested that perhaps CXCL10 produced by astrocytes at the inflamed CNS mostly direct the recruitment of FOXp3+ Tregs that then suppress effector T cells function (37) (Figure 1C). Yet, the authors question the validity and relevance of using CXCL10KO mice, or CXCR3KO mice in EAE studies, as in the absence of CXCL10 produced by astrocyte migration of T cells to the CNS is very limited, and may not reflect the disease in WT mice, or MS patients (37). It should also be noted that vast majority of these experiments were conducted in C57BL/6 mice that lack CXCL11. Finally, Chung & Liao used an adoptive transfer system in which CXCR3^+^ Th17 cells compared to CXCE3^−/−^ Th17 cells were transferred during EAE to suggest that negative signaling via glial cells restrain the activities of Th17 cells within the CNS (42).

In summary, the role of CXCL10 in inflammatory autoimmunity, particularly in neuroinflammation is controversial and need to be further addressed discussed below.

## How the Field Could Move Forward From the Current Controversy?

The controversy of the role of CXCL10 in neuroinflammation, particularly when comparing systemic administration of anti CXCL10 neutralizing antibodies vs. using CXCL10 KO mice should be further addressed, particularly if one would like to consider anti CXCL10 based therapy for autoimmunity. An essential set of experiments should be conducted on CXCR3 KO mice vs. WT and CXCL10 KO mice vs. WT subjected to the induction of different inflammatory autoimmune disease that are not associated with neuroinflammation. Particularly arthritis and IBD. Ideal models would be mice models that express functional CXCL11 (the only one that does not do so is the C57BL/6 mice). Systemic blocked of CXCL10 in various diseases (including neuroinflammation) should be addressed using anti CXCL10 mAbs with very high specificity. Finally, a set-up in which CXCL10 is selectively knocked down from astrocytes would also be helpful for addressing the role of astrocytes CXCL10 in neuroinflammation. An open-end question that should still be unresolved is that why would CXCL10 selectivity recruits FOXp3+ T cells to the CNS?

## Cancer Evolution and Chemokines-Chemokine Receptor Interaction

Chemokine-chemokine receptor interactions play a major role in cancer biology (43–48). The common deterministic dogma suggests along cancer evolution tumor cells evolved to express chemokine receptor and produce their ligands because these interactions support tumor growth by several mechanisms (47, 49–51): First, many of them function as growth/survival factors either by autocrine pathway, and/or by inducing growth factors production by epithelial cells and stromal cells within the tumor microenvironment. Second, several of them direct the recruitment of bone marrow derived cells that support tumor growth and suppress anti-tumor immunity. Third, chemokine—chemokine receptor interactions are involved in attracting tumor cells to metastatic sites. The key chemokine receptor pathways that directly support tumor development are the CCR2-CCL2 (52–56), CXCR4-CXCL12 (48, 57), and CCR5- CCL3/4/5 (58–62) (Table 1). All three pathways are also associated with the recruitment of bone marrow derives cells to the tumor site, and with direct attraction of tumor cells to form metastatic spread. An additional chemokine receptor that recently became of a major interest is CCR8 that is abundant of on FOXp3+ Tregs (63).

**Table 1:** Key chemokine receptor pathways that support tumor development.

**Chemokine receptor -chemokine axis**	**Key pathways**	**References**
CCR2-CCL2	Direct support of tumor growth, recruitment of tumor associated macrophages (TAMs) to support tumor growth and suppress anti-tumor immune reactivity	(52–56)
CXCR4-CXCL12	Direct support of tumor growth, metastatic spread, particularly to the bones	(48, 57)
CCR5- CCL3/4/5	Direct support of tumor growth, recruitment of polymorph nuclear myeloid derived suppressor cells and potentiation of their function at the tumor site.	(58–62)
CCR8-CCL1	CCR8+ Tregs function as master drivers of immune regulation and therefore are key drivers in tumor escape from immune destruction	(63, 64)

Aside of this axis many other chemokine-chemokine receptors are involved in different cancer diseases (for a recent review see (65)). However, the current mini review mostly focuses on CXCR3 and its ligands.

## What is Known About CXCL10 and CXCL9 and in Cancer Immunity?

Several studies showed that CXCL9 and CXCL10, particularly CXCL10 produced by tumor or host cells can recruit CXCR3+ tumor-infiltrating CD4+ T cells, CD8+ T cells and NK cells that are associated with tumor suppression (33, 66–74). Zumwalt et al. showed active secretion of CXCL10 and CCL5 from colorectal cancer microenvironments in human was associates with Granzyme B+ CD8+ T-cell infiltration (75). It is likely that for CD8+ T cells the CXCR3-CXCL10 axis that is involved in directed migration of these cells to the tumor site also induces their potentiation and proliferation there (7, 33) (Figure 1A). What about CXCL9? Very recently Andy Luster and his group showed that anti PD-1 efficacy is reduced in CXCR3KO mice, and suggested that the interaction between CXCL9, largely produced by CD103+ dendritic cells (DC) at the tumor site, and CXCR3 on CD8+ T cells enhances anti PD-1 efficacy (7). The authors also extended this study to humans, suggesting that levels of CXCR3 ligands in the plasma may be used to predict success in anti PD-1 checkpoint therapy (7). It is yet to be explored whether CXCL9 and CXCL10 induce different signaling cascade via CXCR3 in CD8+ T cells.

## What is Known About CXCL10 Based Therapy of Cancer Diseases?

Nineteen years ago, Arenberg et al. showed that intra-tumoral injection of CXCL10 limits non-small-cell lung cancer (NSCLC) in SCID mice by a direct effect on tumor growth (76). Our collaborative study with Israel Vlodavsky was the first to show that systemic administration of CXCL10 (CXCL10-Ig) limits cancer in immunocompetent mice (34). One year later (2015) Peng et al. showed that treatment with epigenetic modulators that increase CXCL9/CXCL10 enhances effector T-cell tumor infiltration, and slows down tumor progression of ovarian cancer (77). At the same year, Barreiara da Silva et al. showed that Dipeptidylpeptidase 4 inhibition enhances endogenous CXCL10 levels and suppresses B16/F10 melanoma growth (78). This study also showed a highly effective effect of Dipeptidylpeptidase 4 based therapy if administered in combination with checkpoint blockers (78). It has recently been suggested that in the set-up of multiple myeloma CXCR3 receptor ligands CXCL9 and CXCL10, limits NK cell positioning into the bone marrow by interfering with CXCR4 function (79). It should also be noted that CXCR3 is expressed on T_regs_ and may be involved in directing their recruitment in cancer and transplantation (11, 12). Collectively this may vote for possible immune-regulating effect. Yet, it has been clearly shown that enhancement of CXCL10 in an *in vivo* set-up increases anti-tumor immunity and could be effectively used for cancer immunotherapy either as monotherapy, or in combined therapy with immune checkpoint inhibitors (78).

## CXCL10 and Brain Cancers

As discussed above chemokines are involved in cancer diseases by several mechanisms among the direct and indirect effect on anti-cancer immunity, direct and indirect effect on cancer growth, and attracting cancer cells to tumor sites. It is generally accepted that CXCL10 enhances anti-cancer immunity, and by so doing limits cancer development. It has also been observed that CXCL10 directly limits cancer (melanoma) growth *in vivo* and *in vitro* (80). Collectively this applies for an anti-cancer property of CXCL10. As for directing metastatic spread Neta Erez and her team very recently suggested that CXCL10 produced by astrocytic cells participates in chemoattraction of tumor cells to the CNS (36). This may give rise to a possible tumor supporting function of CXCL10 in brain metastasis. Nevertheless, as described below many human studies clearly show that in various human cancer diseases low expression/transcription of CXCL10 at tumor sites indicate poor cancer prognosis, whereas high levels of this chemokine are associated with good prognosis.

In summary, CXCL10 is likely to hold anti-cancer propertied that include: 1. Direct effect on the immune system resulting in enhanced anti-cancer response, effect on epithelial cells surrounding the tumor and direct effect on tumor growth. Its tumor supporting role is by attracting tumor cells to form metastasis, as was recently suggested for brain tumors. We are now using CXCR3KO mice engrafted with CXCR3+ tumor cells to dissect the direct effect of CXCL10-Ig based therapy on tumor growth.

## CXCL10 and Canner Prognosis in Human

Ten years ago Jiang et al. reported that low transcription of CXCL10 shows poor prognosis in stage II and III colorectal cancer (81). Later Li et al. showed that in patients with rectal cancer that high expression of CXCL10 may predict better successes in chemoradiotherapy suggesting a synergistic beneficial effect of both (82). Rainczuk et al. showed that high levels of a CXCL10 antagonist in patients with high-grade, serous epithelial ovarian carcinoma (HGSOC) is associated with poor prognosis (83). As for Osteosarcoma (OS), Flores et al. showed better survival in patients with high level of CXCL10 (84). Finally, very recently Zhang et al. showed that in hepatocellular carcinoma (HCC) high levels of CXCL10 are associated with better prognostic and overall survival (85). Several publications challenged this concept (86–88). These studies focused on different cancers: breast cancer, renal cancer and multiple myeloma (86–88). One is that the discrepancy between the studies is because the role of CXCL10 / CXCL9 varies between different cancer disease. If so this should be taken in account as a major criterion in candidate selection for a favorable disease for CXCL10/CXCL9 based therapy.

In summary, CXCL10 is likely to restrict cancer development in many cancers by inducing anti-cancer immune response, and by a direct effect on epithelial cells within the tumor microenvironment and by direct suppression of tumor growth. It is possible that CXCL10 and perhaps pro-cancer function is due to its chemotactic properties for cancer cells.

## Chemoattraction and Beyond, can We Differentially Analyze these Properties?

It is clear that chemoattraction of CXCR3+ T cells, and other CXCR3+ cells, to sites of inflammation and tumor sites is an essential feature, and that inhibition of the CXCR3 dependent migration of CXCR3+ T cells to tumor site, or even their adhesion molecule dependent arrest, plays a major role in inflammation and cancer. For example, Mikucki et al. applied adoptive transfer experiments of T cells from CXCR3KO Vs WT mice in a cancer set-up to show that recruitment to the tumor site was markedly inhibited when donor cells came from CXCR3KO mice, and inhibition was comparable to the one achieved by using T cells form WT donors and pertussis toxin (PTX) (33). It is also clear that CXCL10, and probably CXCL9 signaling enhance the effector properties of these cells (7). We believe that what makes CXCR3 and its ligands drivers of immune function is the combination of chemotaxis and direct effect on the biological function (6, 89). Dissecting the direct effect of CXCR3 ligands on cells migration from their ability to affect the biological properties of these cells could be of interest when developing therapeutic tools, such as blocking antibodies or stabilized chemokines for immunotherapy.

## Conclusions

The main take home message of this minireview is that few chemokine receptors, among them CXCR3, are key drivers in directing the immune response as aside of chemoattraction they also direct the biological function of immune cells that possess them. CXCR3 is of high interest as each of its three ligands differs in its biological properties via this receptor, and its ability to regulate the biological function of others. For example, CXCL11 with the higher affinity to CXCR3 is likely to hold anti-inflammatory properties and by leading to receptor internalization makes the receptor less accessible to others. Currently much attention is given to CXCL9 and CXCL10 and their role in the potentiation of anti-tumor CD8+ T cells.

Chemokine receptors support tumor development different complementary pathways: First, many of them function as growth/survival factors either by autocrine pathway, and/or by inducing growth factors production by epithelial cells and stromal cells within the tumor microenvironment. Second, several of them direct the recruitment of bone marrow derived cells that support tumor growth and suppress anti-tumor immunity. Third, chemokine—chemokine receptor interactions are involved in attracting tumor cells to metastatic sites. Table 1 indicates the involvement of key chemokine receptors in these pathways.

## Author Contributions

The author confirms being the sole contributor of this work and has approved it for publication.

## Conflict of Interest

NK receives from TEVA pharmaceutical company an academic research grant for exploring the role of chemokines in cancer progression (see acknowledgment). The handling Editor declared a past co-authorship with the author.

## References

[1] Luster AD. Chemokines–chemotactic cytokines that mediate inflammation. N Engl J Med. (1998) 338:436–45. 10.1056/NEJM199802123380706 9459648

[2] Luttrell LM Ferguson SS Daaka Y Miller WE Maudsley S Della Rocca GJ. Beta-arrestin-dependent formation of beta2 adrenergic receptor-src protein kinase complexes beta-arrestin-dependent formation of beta2 adrenergic receptor-Src protein kinase complexes. Science. (1999) 283:655–61. 10.1126/science.283.5402.655 9924018

[3] Liu JJ Horst R Katritch V Stevens RC Wuthrich K. Biased signaling pathways in beta2-adrenergic receptor characterized by 19F-NMR. Science. (2012) 335:1106–10. 10.1126/science.1215802 22267580 PMC3292700

[4] Zohar Y Wildbaum G Novak R Salzman AL Thelen M Alon R. CXCL11-dependent induction of FOXP3-negative regulatory T cells suppresses autoimmune encephalomyelitis. J Clin Invest. (2014) 124:2009–22. 10.1172/JCI71951 24713654 PMC4001543

[5] Karin N Wildbaum G Thelen M. Biased signaling pathways via CXCR3 control the development and function of CD4+ T cell subsets. J Leukoc Biol. (2016) 99:857–62. 10.1189/jlb.2MR0915-441R 26657511

[6] Karin N. Chemokines and cancer: new immune checkpoints for cancer therapy. Curr Opin Immunol. (2018) 51:140–5. 10.1016/j.coi.2018.03.004 29579623

[7] Chow MT Ozga AJ Servis RL Frederick DT Lo JA Fisher DE. Intratumoral activity of the CXCR3 chemokine system is required for the efficacy of anti-PD-1 therapy. Immunity. (2019) 50:1498–512.e5. 10.1016/j.immuni.2019.04.010 31097342 PMC6527362

[8] Karpus WJ. Cytokines and chemokines in the pathogenesis of experimental autoimmune encephalomyelitis. J Immunol. (2020) 204:316–26. 10.4049/jimmunol.1900914 31907274

[9] Fenwick PS Macedo P Kilty IC Barnes PJ Donnelly LE. Effect of JAK inhibitors on release of CXCL9, CXCL10 and CXCL11 from human airway epithelial cells. PLoS ONE. (2015) 10:e0128757. 10.1371/journal.pone.0128757 26090665 PMC4474874

[10] Paust HJ Riedel JH Krebs CF Turner JE Brix SR Krohn S. CXCR3+ regulatory T cells control TH1 responses in crescentic GN. J Am Soc Nephrol. (2016) 27:1933–42. 10.1681/ASN.2015020203 26534920 PMC4926966

[11] Li CX Ling CC Shao Y Xu A Li XC Ng KT. CXCL10/CXCR3 signaling mobilized-regulatory T cells promote liver tumor recurrence after transplantation. J Hepatol. (2016) 65:944–52. 10.1016/j.jhep.2016.05.032 27245433

[12] Redjimi N Raffin C Raimbaud I Pignon P Matsuzaki J Odunsi K. CXCR3+ T regulatory cells selectively accumulate in human ovarian carcinomas to limit type I immunity. Cancer Res. (2012) 72:4351–60. 10.1158/0008-5472.CAN-12-0579 22798340

[13] Korniejewska A McKnight AJ Johnson Z Watson ML Ward SG. Expression and agonist responsiveness of CXCR3 variants in human T lymphocytes. Immunology. (2011) 132:503–15. 10.1111/j.1365-2567.2010.03384.x 21255008 PMC3075504

[14] Luster AD Ravetch JV. Biochemical cherecterization of gamma interferon inducible cytokine (IP-10). J Exp Med. (1987) 166:1084–97. 10.1084/jem.166.4.1084 2443596 PMC2188708

[15] Cole KE Strick CA Paradis TJ Ogborne KT Loetscher M Gladue RP. Interferon-inducible T cell alpha chemoattractant (I-TAC): a novel non-ELR CXC chemokine with potent activity on activated T cells through selective high affinity binding to CXCR3. J Exp Med. (1998) 187:2009–21. 10.1084/jem.187.12.2009 9625760 PMC2212354

[16] Abbas AK Murphy KM Sher A. Functional diversity of helper T lymphocytes. Nature. (1996) 383:787–93. 10.1038/383787a0 8893001

[17] Youssef S Maor G Wildbaum G Grabie N Gour-Lavie A Karin N. C-C chemokine-encoding DNA vaccines enhance breakdown of tolerance to their gene products and treat ongoing adjuvant arthritis. J Clin Invest. (2000) 106:361–71. 10.1172/JCI9109 10930439 PMC314324

[18] Youssef S Wildbaum G Maor G Lanir N Gour-Lavie A Grabie N. Long-lasting protective immunity to experimental autoimmune encephalomyelitis following vaccination with naked DNA encoding C-C chemokines. J Immunol. (1998) 161:3870–9. 9780152

[19] Wildbaum G Nahir MA Karin N. Beneficial autoimmunity to proinflammatory mediators restrains the consequences of self-destructive immunity. Immunity. (2003) 19:679–88. 10.1016/S1074-7613(03)00291-7 14614855

[20] Wildbaum G Netzer N Karin N. Plasmid DNA encoding IFN-gamma-inducible protein 10 redirects antigen-specific T cell polarization and suppresses experimental autoimmune encephalomyelitis. J Immunol. (2002) 168:5885–92. 10.4049/jimmunol.168.11.5885 12023393

[21] Salomon I Netzer N Wildbaum G Schif-Zuck S Maor G Karin N. Targeting the function of IFN-gamma-inducible protein 10 suppresses ongoing adjuvant arthritis. J Immunol. (2002) 169:2685–93. 10.4049/jimmunol.169.5.2685 12193742

[22] Gangur V Simons FE Hayglass KT. Human IP-10 selectively promotes dominance of polyclonally activated and environmental antigen-driven IFN-gamma over IL-4 responses. FASEB J. (1998) 12:705–13. 10.1096/fasebj.12.9.705 9619449

[23] Wildbaum G Westermann J Maor G Karin N. A targeted DNA vaccine encoding fas ligand defines its dual role in the regulation of experimental autoimmune encephalomyelitis. J Clin Invest. (2000) 106:671–9. 10.1172/JCI8759 10974020 PMC381283

[24] Sierro F Biben C Martinez-Munoz L Mellado M Ransohoff RM Li M. Disrupted cardiac development but normal hematopoiesis in mice deficient in the second CXCL12/SDF-1 receptor, CXCR7. Proc Natl Acad Sci USA. (2007) 104:14759–64. 10.1073/pnas.0702229104 17804806 PMC1976222

[25] Samama P Cotecchia S Costa T Lefkowitz RJ. A mutation-induced activated state of the beta 2-adrenergic receptor. Extending the ternary complex model. J Biol Chem. (1993) 268:4625–36. 8095262

[26] Reiter E Ahn S Shukla AK Lefkowitz RJ. Molecular mechanism of beta-arrestin-biased agonism at seven-transmembrane receptors. Annu Rev Pharmacol Toxicol. (2012) 52:179–97. 10.1146/annurev.pharmtox.010909.105800 21942629 PMC3628752

[27] Kim J Choi JY Park SH Yang SH Park JA Shin K. Therapeutic effect of anti-C-X-C motif chemokine 10 (CXCL10) antibody on C protein-induced myositis mouse. Arthritis Res Ther. (2014) 16:R126. 10.1186/ar4583 24939012 PMC4095607

[28] Yellin M Paliienko I Balanescu A Ter-Vartanian S Tseluyko V Xu LA. A phase II, randomized, double-blind, placebo-controlled study evaluating the efficacy and safety of MDX-1100, a fully human anti-CXCL10 monoclonal antibody, in combination with methotrexate in patients with rheumatoid arthritis. Arthritis Rheum. (2012) 64:1730–9. 10.1002/art.34330 22147649

[29] Singh UP Singh S Singh R Cong Y Taub DD Lillard JW Jr. CXCL10-producing mucosal CD4+ T cells, NK cells, and NKT cells are associated with chronic colitis in IL-10(-/-) mice, which can be abrogated by anti-CXCL10 antibody inhibition. J Interferon Cytokine Res. (2008) 28:31–43. 10.1089/jir.2007.0059 18370870 PMC2435497

[30] Christen U McGavern DB Luster AD von Herrath MG Oldstone MB. Among CXCR3 chemokines, IFN-gamma-inducible protein of 10 kDa (CXC chemokine ligand (CXCL) 10) but not monokine induced by IFN-gamma (CXCL9) imprints a pattern for the subsequent development of autoimmune disease. J Immunol. (2003) 171:6838–45. 10.4049/jimmunol.171.12.6838 14662890

[31] Frigerio S Junt T Lu B Gerard C Zumsteg U Hollander GA. Beta cells are responsible for CXCR3-mediated T-cell infiltration in insulitis. Nat Med. (2002) 8:1414–20. 10.1038/nm1202-792 12415259

[32] Kuo PT Zeng Z Salim N Mattarollo S Wells JW Leggatt GR. The role of CXCR3 and its chemokine ligands in skin disease and cancer. Front Med (Lausanne). (2018) 5:271. 10.3389/fmed.2018.00271 30320116 PMC6167486

[33] Mikucki ME Fisher DT Matsuzaki J Skitzki JJ Gaulin NB Muhitch JB. Non-redundant requirement for CXCR3 signalling during tumoricidal T-cell trafficking across tumour vascular checkpoints. Nat Commun. (2015) 6:7458. 10.1038/ncomms8458 26109379 PMC4605273

[34] Barash U Zohar Y Wildbaum G Beider K Nagler A Karin N. Heparanase enhances myeloma progression via CXCL10 downregulation. Leukemia. (2014) 28:2178–87. 10.1038/leu.2014.121 24699306 PMC4185261

[35] Nagpal ML Davis J Lin T. Overexpression of CXCL10 in human prostate LNCaP cells activates its receptor (CXCR3) expression and inhibits cell proliferation. Biochim Biophys Acta. (2006) 1762:811–8. 10.1016/j.bbadis.2006.06.017 16934957

[36] Doron H Amer M Ershaid N Blazquez R Shani O Lahav TG. Inflammatory activation of astrocytes facilitates melanoma brain tropism via the CXCL10-CXCR3 signaling axis. Cell Rep. (2019) 28:1785–98.e6. 10.1016/j.celrep.2019.07.033 31412247

[37] Muller M Carter S Hofer MJ Campbell IL. Review: the chemokine receptor CXCR3 and its ligands CXCL9, CXCL10 and CXCL11 in neuroimmunity–a tale of conflict and conundrum. Neuropathol Appl Neurobiol. (2010) 36:368–87. 10.1111/j.1365-2990.2010.01089.x 20487305

[38] Muller M Carter SL Hofer MJ Manders P Getts DR Getts MT. CXCR3 signaling reduces the severity of experimental autoimmune encephalomyelitis by controlling the parenchymal distribution of effector and regulatory T cells in the central nervous system. J Immunol. (2007) 179:2774–86. 10.4049/jimmunol.179.5.2774 17709491

[39] Fife BT Kennedy KJ Paniagua MC Lukacs NW Kunkel SL Luster AD. CXCL10 (IFN-gamma-inducible protein-10) control of encephalitogenic CD4+ T cell accumulation in the central nervous system during experimental autoimmune encephalomyelitis. J Immunol. (2001) 166:7617–24. 10.4049/jimmunol.166.12.7617 11390519

[40] Liu L Huang D Matsui M He TT Hu T Demartino J. Severe disease, unaltered leukocyte migration, and reduced IFN-gamma production in CXCR3-/- mice with experimental autoimmune encephalomyelitis. J Immunol. (2006) 176:4399–409. 10.4049/jimmunol.176.7.4399 16547278

[41] Klein RS Izikson L Means T Gibson HD Lin E Sobel RA. IFN-inducible protein 10/CXC chemokine ligand 10-independent induction of experimental autoimmune encephalomyelitis. J Immunol. (2004) 172:550–9. 10.4049/jimmunol.172.1.550 14688366

[42] Chung CY Liao F. CXCR3 signaling in glial cells ameliorates experimental autoimmune encephalomyelitis by restraining the generation of a pro-Th17 cytokine milieu and reducing CNS-infiltrating Th17 cells. J Neuroinflammation. (2016) 13:76. 10.1186/s12974-016-0536-4 27068264 PMC4828793

[43] Amit M Na'ara S Gil Z. Mechanisms of cancer dissemination along nerves. Nat Rev Cancer. (2016) 16:399–408. 10.1038/nrc.2016.38 27150016

[44] Balkwill F. Cancer and the chemokine network. Nat Rev Cancer. (2004) 4:540–50. 10.1038/nrc1388 15229479

[45] Murdoch C Muthana M Coffelt SB Lewis CE. The role of myeloid cells in the promotion of tumour angiogenesis. Nat Rev Cancer. (2008) 8:618–31. 10.1038/nrc2444 18633355

[46] Homey B Muller A Zlotnik A. Chemokines: agents for the immunotherapy of cancer? Nat Rev Immunol. (2002) 2:175–84. 10.1038/nri748 11913068

[47] Nagarsheth N Wicha MS Zou W. Chemokines in the cancer microenvironment and their relevance in cancer immunotherapy. Nat Rev Immunol. (2017) 17:559–72. 10.1038/nri.2017.49 28555670 PMC5731833

[48] Zlotnik A Burkhardt AM Homey B. Homeostatic chemokine receptors and organ-specific metastasis. Nat Rev Immunol. (2011) 11:597–606. 10.1038/nri3049 21866172

[49] Dart A. Tumour microenvironment: radical changes. Nat Rev Cancer. (2018) 18:65. 10.1038/nrc.2018.4 29368744

[50] Binnewies M Roberts EW Kersten K Chan V Fearon DF Merad M. Understanding the tumor immune microenvironment (TIME) for effective therapy. Nat Med. (2018) 24:541–50. 10.1038/s41591-018-0014-x 29686425 PMC5998822

[51] Danovi S. Tumour microenvironment: as time goes by. Nat Rev Cancer. (2016) 16:342–3. 10.1038/nrc.2016.53 27150015

[52] Loberg RD Ying C Craig M Day LL Sargent E Neeley C. Targeting CCL2 with systemic delivery of neutralizing antibodies induces prostate cancer tumor regression *in vivo*. Cancer Res. (2007) 67:9417–24. 10.1158/0008-5472.CAN-07-1286 17909051

[53] Conti I Rollins BJ. CCL2 (monocyte chemoattractant protein-1) and cancer. Semin Cancer Biol. (2004) 14:149–54. 10.1016/j.semcancer.2003.10.009 15246049

[54] Qian BZ Li J Zhang H Kitamura T Zhang J Campion LR. CCL2 recruits inflammatory monocytes to facilitate breast-tumour metastasis. Nature. (2011) 475:222–5. 10.1038/nature10138 21654748 PMC3208506

[55] Izhak L Wildbaum G Jung S Stein A Shaked Y Karin N. Dissecting the autocrine and paracrine roles of the CCR2-CCL2 axis in tumor survival and angiogenesis. PLoS ONE. (2012) 7:e28305. 10.1371/journal.pone.0028305 22279523 PMC3261135

[56] Sica A Saccani A Bottazzi B Bernasconi S Allavena P Gaetano B. Defective expression of the monocyte chemotactic protein-1 receptor CCR2 in macrophages associated with human ovarian carcinoma. J Immunol. (2000) 164:733–8. 10.4049/jimmunol.164.2.733 10623817

[57] Staller P Sulitkova J Lisztwan J Moch H Oakeley EJ Krek W. Chemokine receptor CXCR4 downregulated by von hippel-lindau tumour suppressor pVHL. Nature. (2003) 425:307–11. 10.1038/nature01874 13679920

[58] Blattner C Fleming V Weber R Himmelhan B Altevogt P Gebhardt C. CCR5(+) myeloid-derived suppressor cells are enriched and activated in melanoma lesions. Cancer Res. (2018) 78:157–67. 10.1158/0008-5472.CAN-17-0348 29089297

[59] Hawila E Razon H Wildbaum G Blattner C Sapir Y Shaked Y. CCR5 directs the mobilization of CD11b(+)Gr1(+)Ly6C(low) polymorphonuclear myeloid cells from the bone marrow to the blood to support tumor development. Cell Rep. (2017) 21:2212–22. 10.1016/j.celrep.2017.10.104 29166611

[60] Halama N Zoernig I Berthel A Kahlert C Klupp F Suarez-Carmona M. Tumoral immune cell exploitation in colorectal cancer metastases can be targeted effectively by anti-CCR5 therapy in cancer patients. Cancer Cell. (2016) 29:587–601. 10.1016/j.ccell.2016.03.005 27070705

[61] Velasco-Velazquez M Pestell RG. The CCL5/CCR5 axis promotes metastasis in basal breast cancer. Oncoimmunology. (2013) 2:e23660. 10.4161/onci.23660 23734321 PMC3654591

[62] Velasco-Velazquez M Jiao X De La Fuente M Pestell TG Ertel A Lisanti MP. CCR5 antagonist blocks metastasis of basal breast cancer cells. Cancer Res. (2012) 72:3839–50. 10.1158/0008-5472.CAN-11-3917 22637726

[63] Barsheshet Y Wildbaum G Levy E Vitenshtein A Akinseye C Griggs J. CCR8(+)FOXp3(+) treg cells as master drivers of immune regulation. Proc Natl Acad Sci USA. (2017) 114:6086–91. 10.1073/pnas.1621280114 28533380 PMC5468670

[64] Plitas G Konopacki C Wu K Bos PD Morrow M Putintseva EV. Regulatory T cells exhibit distinct features in human breast cancer. Immunity. (2016) 45:1122–34. 10.1016/j.immuni.2016.10.032 27851913 PMC5134901

[65] Jacquelot N Duong CPM Belz GT Zitvogel L. Targeting chemokines and chemokine receptors in melanoma and other cancers. Front Immunol. (2018) 9:2480. 10.3389/fimmu.2018.02480 30420855 PMC6215820

[66] Harlin H Meng Y Peterson AC Zha Y Tretiakova M Slingluff C. Chemokine expression in melanoma metastases associated with CD8+ T-cell recruitment. Cancer Res. (2009) 69:3077–85. 10.1158/0008-5472.CAN-08-2281 19293190 PMC3886718

[67] Luster AD Leder P IP-10 a-C-X-C- chemokine elicits a potent thymus-dependent antitumor response *in vivo*. J Exp Med. (1993) 178:1057–65. 10.1084/jem.178.3.1057 8350046 PMC2191174

[68] Fujita M Zhu X Ueda R Sasaki K Kohanbash G Kastenhuber ER. Effective immunotherapy against murine gliomas using type 1 polarizing dendritic cells–significant roles of CXCL10. Cancer Res. (2009) 69:1587–95. 10.1158/0008-5472.CAN-08-2915 19190335 PMC5450639

[69] Wennerberg E Kremer V Childs R Lundqvist A. CXCL10-induced migration of adoptively transferred human natural killer cells toward solid tumors causes regression of tumor growth *in vivo*. Cancer Immunol Immunother. (2015) 64:225–35. 10.1007/s00262-014-1629-5 25344904 PMC11028951

[70] Wenzel J Bekisch B Uerlich M Haller O Bieber T Tuting T. Type I interferon-associated recruitment of cytotoxic lymphocytes: a common mechanism in regressive melanocytic lesions. Am J Clin Pathol. (2005) 124:37–48. 10.1309/4EJ9KL7CGDENVVLE 15923172

[71] de Lange MJ Nell RJ Lalai RN Versluis M Jordanova ES Luyten GPM. Digital PCR-based T-cell quantification-assisted deconvolution of the microenvironment reveals that activated macrophages drive tumor inflammation in uveal melanoma. Mol Cancer Res. (2018) 16:1902–11. 10.1158/1541-7786.MCR-18-0114 30093564

[72] Dengel LT Norrod AG Gregory BL Clancy-Thompson E Burdick MD Strieter RM. Interferons induce CXCR3-cognate chemokine production by human metastatic melanoma. J Immunother. (2010) 33:965–74. 10.1097/CJI.0b013e3181fb045d 20948440 PMC3110268

[73] Kunz M Toksoy A Goebeler M Engelhardt E Brocker E Gillitzer R. Strong expression of the lymphoattractant C-X-C chemokine mig is associated with heavy infiltration of T cells in human malignant melanoma. J Pathol. (1999) 189:552–8. 10.1002/(SICI)1096-9896(199912)189:4<552::AID-PATH469>3.0.CO;2-I 10629557

[74] Ganju RK Brubaker SA Meyer J Dutt P Yang Y Qin S. The alpha-chemokine, stromal cell-derived factor-1alpha, binds to the transmembrane G-protein-coupled CXCR-4 receptor and activates multiple signal transduction pathways. J Biol Chem. (1998) 273:23169–75. 10.1074/jbc.273.36.23169 9722546

[75] Zumwalt TJ Arnold M Goel A Boland CR. Active secretion of CXCL10 and CCL5 from colorectal cancer microenvironments associates with GranzymeB+ CD8+ T-cell infiltration. Oncotarget. (2015) 6:2981–91. 10.18632/oncotarget.3205 25671296 PMC4413778

[76] Arenberg DA White ES Burdick MD Strom SR Strieter RM. Improved survival in tumor-bearing SCID mice treated with interferon-gamma-inducible protein 10 (IP-10/CXCL10). Cancer Immunol Immunother. (2001) 50:533–8. 10.1007/s00262-001-0231-9 11776375 PMC11032922

[77] Peng D Kryczek I Nagarsheth N Zhao L Wei S Wang W. Epigenetic silencing of TH1-type chemokines shapes tumour immunity and immunotherapy. Nature. (2015) 527:249–53. 10.1038/nature15520 26503055 PMC4779053

[78] da Silva RB Laird ME Yatim N Fiette L Ingersoll MA Albert ML Dipeptidylpeptidase 4 inhibition enhances lymphocyte trafficking, improving both naturally occurring tumor immunity and immunotherapy. Nat Immunol. (2015) 16:850–8. 10.1038/ni.3201 26075911

[79] Bonanni V Antonangeli F Santoni A Bernardini G. Targeting of CXCR3 improves anti-myeloma efficacy of adoptively transferred activated natural killer cells. J Immunother Cancer. (2019) 7:290. 10.1186/s40425-019-0751-5 31699153 PMC6839099

[80] Antonicelli F Lorin J Kurdykowski S Gangloff SC Le Naour R Sallenave JM. CXCL10 reduces melanoma proliferation and invasiveness *in vitro* and *in vivo*. Br J Dermatol. (2011) 164:720–8. 10.1111/j.1365-2133.2010.10176.x 21155750

[81] Jiang Z Xu Y Cai S. CXCL10 expression and prognostic significance in stage II and III colorectal cancer. Mol Biol Rep. (2010) 37:3029–36. 10.1007/s11033-009-9873-z 19821051

[82] Li C Wang Z Liu F Zhu J Yang L Cai G. CXCL10 mRNA expression predicts response to neoadjuvant chemoradiotherapy in rectal cancer patients. Tumour Biol. (2014) 35:9683–91. 10.1007/s13277-014-2234-0 24969558

[83] Rainczuk A Rao JR Gathercole JL Fairweather NJ Chu S Masadah R. Evidence for the antagonistic form of CXC-motif chemokine CXCL10 in serous epithelial ovarian tumours. Int J Cancer. (2014) 134:530–41. 10.1002/ijc.28393 23873303

[84] Flores RJ Kelly AJ Li Y Nakka M Barkauskas DA Krailo M. A novel prognostic model for osteosarcoma using circulating CXCL10 and FLT3LG. Cancer. (2017) 123:144–54. 10.1002/cncr.30272 27529817 PMC5161556

[85] Zhang J Chen J Guan GW Zhang T Lu FM Chen XM. [Expression and clinical significance of chemokine CXCL10 and its receptor CXCR3 in hepatocellular carcinoma]. Beijing Da Xue Xue Bao Yi Xue Ban. (2019) 51:402–8. 10.19723/j.issn.1671-167X.2019.03.005 31209409 PMC7439049

[86] Bolomsky A Schreder M Hubl W Zojer N Hilbe W Ludwig H. Monokine induced by interferon gamma (MIG/CXCL9) is an independent prognostic factor in newly diagnosed myeloma. Leuk Lymphoma. (2016) 57:2516–25. 10.3109/10428194.2016.1151511 26999330

[87] Lunardi S Jamieson NB Lim SY Griffiths KL Carvalho-Gaspar M Al-Assar O. IP-10/CXCL10 induction in human pancreatic cancer stroma influences lymphocytes recruitment and correlates with poor survival. Oncotarget. (2014) 5:11064–80. 10.18632/oncotarget.2519 25415223 PMC4294325

[88] Mulligan AM Raitman I Feeley L Pinnaduwage D Nguyen LT O'Malley FP. Tumoral lymphocytic infiltration and expression of the chemokine CXCL10 in breast cancers from the ontario familial breast cancer registry. Clin Cancer Res. (2013) 19:336–46. 10.1158/1078-0432.CCR-11-3314 23213058 PMC3548938

[89] Karin N Razon H. Chemokines beyond chemo-attraction: CXCL10 and its significant role in cancer and autoimmunity. Cytokine. (2018) 109:24–8. 10.1016/j.cyto.2018.02.012 29449068

